# 

**Published:** 1923-01

**Authors:** 


### Contents

Event and Comment .................... 1
American Dental Society of Europe .................... 3
Some Characteristics of the Tissues Composing and Surrounding the Tooth-root Applicable in the Prevention and Treatment of Infections .................... 10
Rationalism vs. Radicalism .................... 28
Dental Clinics in Public Institutions and in Public Schools... 34
Books Received and Reviewed .................... 39
Indents .................... 44
Announcements .................... 47

